# Continuous Digital Monitoring of Walking Speed in Frail Elderly Patients: Noninterventional Validation Study and Longitudinal Clinical Trial

**DOI:** 10.2196/15191

**Published:** 2019-11-27

**Authors:** Arne Mueller, Holger Alfons Hoefling, Amir Muaremi, Jens Praestgaard, Lorcan C Walsh, Ola Bunte, Roland Martin Huber, Julian Fürmetz, Alexander Martin Keppler, Matthias Schieker, Wolfgang Böcker, Ronenn Roubenoff, Sophie Brachat, Daniel S Rooks, Ieuan Clay

**Affiliations:** 1 Novartis Institutes for BioMedical Research Basel Switzerland; 2 Biostatistics and Pharmacometrics Novartis Pharmaceuticals Corporation East Hannover, NJ United States; 3 Novartis Business Services Novartis Ireland Ltd Dublin Ireland; 4 University Hospital Ludwigs-Maximillians Universität Munich Germany; 5 Novartis Institutes for BioMedical Research Cambridge, MA United States

**Keywords:** gait, walking speed, mobility limitation, accelerometry, clinical trials, frailty, wearable electronic devices, algorithms, open source data, data collection, dataset

## Abstract

**Background:**

Digital technologies and advanced analytics have drastically improved our ability to capture and interpret health-relevant data from patients. However, only limited data and results have been published that demonstrate accuracy in target indications, real-world feasibility, or the validity and value of these novel approaches.

**Objective:**

This study aimed to establish accuracy, feasibility, and validity of continuous digital monitoring of walking speed in frail, elderly patients with sarcopenia and to create an open source repository of raw, derived, and reference data as a resource for the community.

**Methods:**

Data described here were collected as a part of 2 clinical studies: an independent, noninterventional validation study and a phase 2b interventional clinical trial in older adults with sarcopenia. In both studies, participants were monitored by using a waist-worn inertial sensor. The cross-sectional, independent validation study collected data at a single site from 26 naturally slow-walking elderly subjects during a parcours course through the clinic, designed to simulate a real-world environment. In the phase 2b interventional clinical trial, 217 patients with sarcopenia were recruited across 32 sites globally, where patients were monitored over 25 weeks, both during and between visits.

**Results:**

We have demonstrated that our approach can capture in-clinic gait speed in frail slow-walking adults with a residual standard error of 0.08 m per second in the independent validation study and 0.08, 0.09, and 0.07 m per second for the 4 m walk test (4mWT), 6-min walk test (6MWT), and 400 m walk test (400mWT) standard gait speed assessments, respectively, in the interventional clinical trial. We demonstrated the feasibility of our approach by capturing 9668 patient-days of real-world data from 192 patients and 32 sites, as part of the interventional clinical trial. We derived inferred contextual information describing the length of a given walking bout and uncovered positive associations between the short 4mWT gait speed assessment and gait speed in bouts between 5 and 20 steps (correlation of 0.23) and longer 6MWT and 400mWT assessments with bouts of 80 to 640 steps (correlations of 0.48 and 0.59, respectively).

**Conclusions:**

This study showed, for the first time, accurate capture of real-world gait speed in slow-walking older adults with sarcopenia. We demonstrated the feasibility of long-term digital monitoring of mobility in geriatric populations, establishing that sufficient data can be collected to allow robust monitoring of gait behaviors outside the clinic, even in the absence of feedback or incentives. Using inferred context, we demonstrated the ecological validity of in-clinic gait assessments, describing positive associations between in-clinic performance and real-world walking behavior. We make all data available as an open source resource for the community, providing a basis for further study of the relationship between standardized physical performance assessment and real-world behavior and independence.

## Introduction

### Background

Gait speed is considered a key prognostic marker of survival [1] and adverse events [2] in older adults and has been shown to decline over time with healthy aging [3]. These observations have primarily been driven by data collected as part of controlled-environment performance tests, and we still understand very little in terms of how performance in the clinic relates to behavior in the real world, although it is clear that these represent distinct, but related, aspects of function as related to mobility [4].

Digital sensor technologies have drastically improved our ability to capture health-relevant data from patients, in particular, data describing real-world behaviors [5]. However, progress on interpretation via advanced analytics is undermined by a lack of algorithms with validated accuracy and performance in disease populations [6], and most clinical trials still focus exclusively on established performance tests.

### Objectives

Age-related muscle loss and weakness (ie, sarcopenia [7]) can result in an accelerated loss of patient mobility, progressive limitations in independence, and reduction in health-related quality of life (HRQoL). Developing therapies with the potential to maintain and improve real-world patient mobility is therefore a critical need. Previous early phase studies have demonstrated the therapeutic potential of increasing muscle mass in patients with sarcopenia [8]. To explore real-world functional consequences associated with changes in ability captured by clinical assessments, we incorporated continuous patient monitoring using a wearable inertial sensor, during and between their planned clinical site visits in the next clinical phase.

The work presented here describes progress on (1) ensuring that we are accurately capturing gait speed in our target population, (2) the feasibility of deployment in a global clinical trial, and (3) comparison with established gait speed performance measures to explore the validity of this novel, digital, continuous monitoring approach.

We hypothesized that this approach would allow us to examine real-world mobility on an individual patient basis and explore the relationship between currently accepted measures of mobility and real-world mobility behaviors, laying a foundation for measuring personalized response to therapy in a way that was not previously possible using in-clinic tests.

## Methods

Data described here were collected from 2 clinical studies: an independent, noninterventional validation study and a phase 2b interventional clinical trial in older adults with sarcopenia.

### Study Design: Independent Validation Study

The independent validation study was performed between May and August 2018 as a cross-sectional design at a single site, with no pharmaceutical treatment, where 26 naturally slow-walking elderly subjects were recruited in 4 cohorts based on their baseline self-selected gait speed over 4 m (4 m walk test, 4mWT): below 0.5 m per second, 0.5 to 0.6 m per second, 0.6 to 0.7 m per second, and 0.7 to 0.8 m per second. Subjects whose walking speed and natural movement were restricted by orthopedic or neurological complications or other relevant medical conditions were not eligible for the study. Data were recorded with a wearable inertial sensor (see section Accelerometry) while the subjects completed a *parcours* course, at least twice, through the clinic that was designed to simulate a real-world environment [9]. The parcours course included straight corridors, stairs, a ramp, and a flat outdoor section. Reference walking speeds were simultaneously measured using a novel combination of a standard distance measuring wheel and an inertial sensor, operated by an assistant, allowing correlation analyses between the patient’s actual speed and the algorithm-derived gait speed estimation [10]. A standard smartphone video device on the measuring wheel recorded the subject’s footfall in slow motion, allowing a more detailed analysis of different sections of the parcours course. A summary of the demographic data of the subjects in the validation study is given in Table 1. Raw and derived data as well as annotations and metadata captured during the study are made available as delimited text files (see section *How to Access*).

**Table 1:** Summaries of demographic data for subjects included in the independent validation study.

Demographic data		Values
Total subjects enrolled, n		26
**Gender, n**		
	Male	11
	Female	15
**Age (years), n (%)**		
	60-65	2 (8)
	66-75	4 (15)
	76-89	18 (69)
	>89	2 (8)
4 m walk test gait speed at enrolment in meter per second, mean (SD)		0.62 (0.12)

### Study Design: Interventional Clinical Trial

The phase 2b, interventional clinical trial (ClinicalTrials.gov identifier: NCT02333331 [11]) recruited 217 patients across 32 sites globally, based on criteria for their lean muscle mass, age, grip strength, and gait speed [12]. Between January 2015 and December 2018, patients were enrolled for a period of 25 weeks each, with clinical assessments occurring across 8 visits. Accelerometry data were collected from patients during clinical gait assessments at baseline, at weeks 9, 17, and 25, and during intervening periods at home. A summary of the demographic data of the patients and of accelerometry and clinical assessment data collected in the interventional clinical trial is provided in Table 2.

At each clinical site visit, patients completed several clinical assessments including a 4mWT [13], 6-min walk test (6MWT [14]), and 400 m walk test (400mWT [15]) from which we derived average walking speed. For each of these assessments, accelerometry data were recorded, along with accurate timestamps delimiting when the patient started and finished each assessment.

Data captured during the trial and metadata describing the assessments are made available as delimited text files (see section *How to Access*).

**Table 2:** A summary of demographic data for patients included in the interventional clinical trial.

Demographic data		Values
Total patients enrolled, n		217
Total patients with accelerometry data, n		192
**Gender, n**		
	Male	91
	Female	126
Age (years), mean (SD)		79.0 (5.45)
4 m walk test gait speed at enrolment, mean (SD)		0.648 (0.1048)

### Accelerometry

For both studies, participants were monitored using a waist-worn inertial sensor (actibelt RCT2, Trium Analysis Online, Munich [3,6,16,17]), which recorded acceleration in 3 dimensions at a sampling frequency of 100 Hz (12-bit resolution) and a range of 6g. The devices did not require charging or other interaction from the patients and were otherwise self-managed by the patients.

For the independent validation study, subjects wore a single device for the duration of their single-visit assessment.

In the interventional clinical trial, each patient was instructed to wear the device continuously for a minimum period of 5 days before each planned clinical site visit, and in cases of self-reported noncompliance, for a further 5 days following the visit. Multiple devices, each of the same model, design, and placement, were used over the observation period. Recorded data were stored locally on the device and downloaded periodically following exchange of devices at planned clinical site visits. Resulting recordings were then merged to form a single observation period. In total, 9668 patient-days of data were collected from 192 patients across 32 sites.

All files are made available in the HDF5 format (see section *How to Access*).

### Derived Gait Parameters and Annotations

Furthermore, algorithmically derived, aggregated gait parameters were calculated for periods when the patients or subjects were actively performing clinical assessments and for periods when patients were passively monitored in their real-world home environment. Individual raw acceleration data files for a given patient were processed using an algorithm similar to that used in a study by Sabatini et al [18], where steps are first detected and parameterized before a Hilbert transform is used to calculate an analytical signal from which gait speed is projected per step using a linear model. In the first step, a short-time Fourier transform is used to extract dominant frequencies from the raw signal: a broad band (0.7-3 Hz) filter pass removes some noise from the signal before it is divided into overlapping windows of approximately 2.5 seconds; and a fast Fourier transform then calculates the frequency domain for each axis. For each window, these results are then combined to determine the dominant frequencies, removing windows where the angle toward gravity or overall activity is not plausible for upright walking or where there is no dominant frequency. This ensures that false-positive (ie, nonwalking) motions are removed. For windows that pass these checks, a Butterworth filter is applied, and a Hilbert transform is used to determine the frequency (*F*), phase (), and amplitude (*A*) for each axis (vertical, *x*; lateral, *y*; and longitudinal, *z*). *A_x_*, *A_y_*, and *A_z_* give good indication of the force involved in a step, independent of the exact time point, whereas *F* indicates step frequency and () yields the relative position within a step. Finally, to predict gait speed, a linear model is fit to the parameters *A_x_*, *A_y_*, and *A_z_* and their interaction terms. By combining information from all 3 axes, this approach allows for much improved step detection in slow, frail walkers, compared with previous methods [9].

To allow fair comparisons over time, periods of continuous walking (*bouts*) were grouped according to length, similar to our previous work [16]. To achieve this, contiguous windows were combined into a single bout, provided that the mean vertical frequency (*F_x_*) did not vary by more than an empirical factor of 1.6-fold between windows.

Derived data describing real-world and in-clinic gait parameters are made available as delimited text (daily summaries and in-clinic assessments) or HDF5 (step- and bout-level summaries) files.

### How to Access

Written informed consent was obtained from the patients for collection and use of the data, and a local ethics committee waiver was granted for collection and publication of the datasets. Anonymized, open source derived data and metadata for the independent validation study [19] and interventional clinical trial [20] are made available. Full datasets, including raw accelerometry data, for both studies are also available for download [21].

### Statement of Ethics

The ethics committee of the Medical Faculty of the Ludwig Maximilian University of Munich (Ref. 17-798) approved the independent validation study. Individual local ethics approval was obtained for all sites in the interventional clinical trial (ClinicalTrials.gov identifier: NCT02333331).

## Results

### Accurate Monitoring of Real-World Gait Speed in Slow-Walking, Older Adults in Controlled Settings

Previous work has shown effective gait speed monitoring in healthy populations by algorithms combining individual detection and parameterization of steps with estimation of gait speed for a given step. Step detection has been achieved with a range of methods including continuous wavelet transform [16,22], whereas gait speed estimation is typically done by using supervised methods such as support vector regression [3]. However, when applied to slow-walking populations, for example, multiple sclerosis (MS), these algorithms produce an observable overestimation of gait speed, particularly in the slowest walkers [6], presumably because of varying relevance of the feature set in patients with pathological gait relative to training sets. The step detector described in our study (see section Methods) is specifically tailored to achieve excellent step detection performance in slow and frail walkers [9]. To validate the accuracy of this new algorithm, in our target population, we performed an independent validation study comparing gait speed continuously captured via an assistant-operated device [10] with gait speed estimated from accelerometry data. The study collected data from 26 frail, elderly adults across a *parcours* course, which simulated a range of real-world situations including indoor corridors, stairs, and a ramp as well as a short outdoor section on uneven ground [9]. A summary of study enrolment is provided in Table 1.

**Figure 1:**
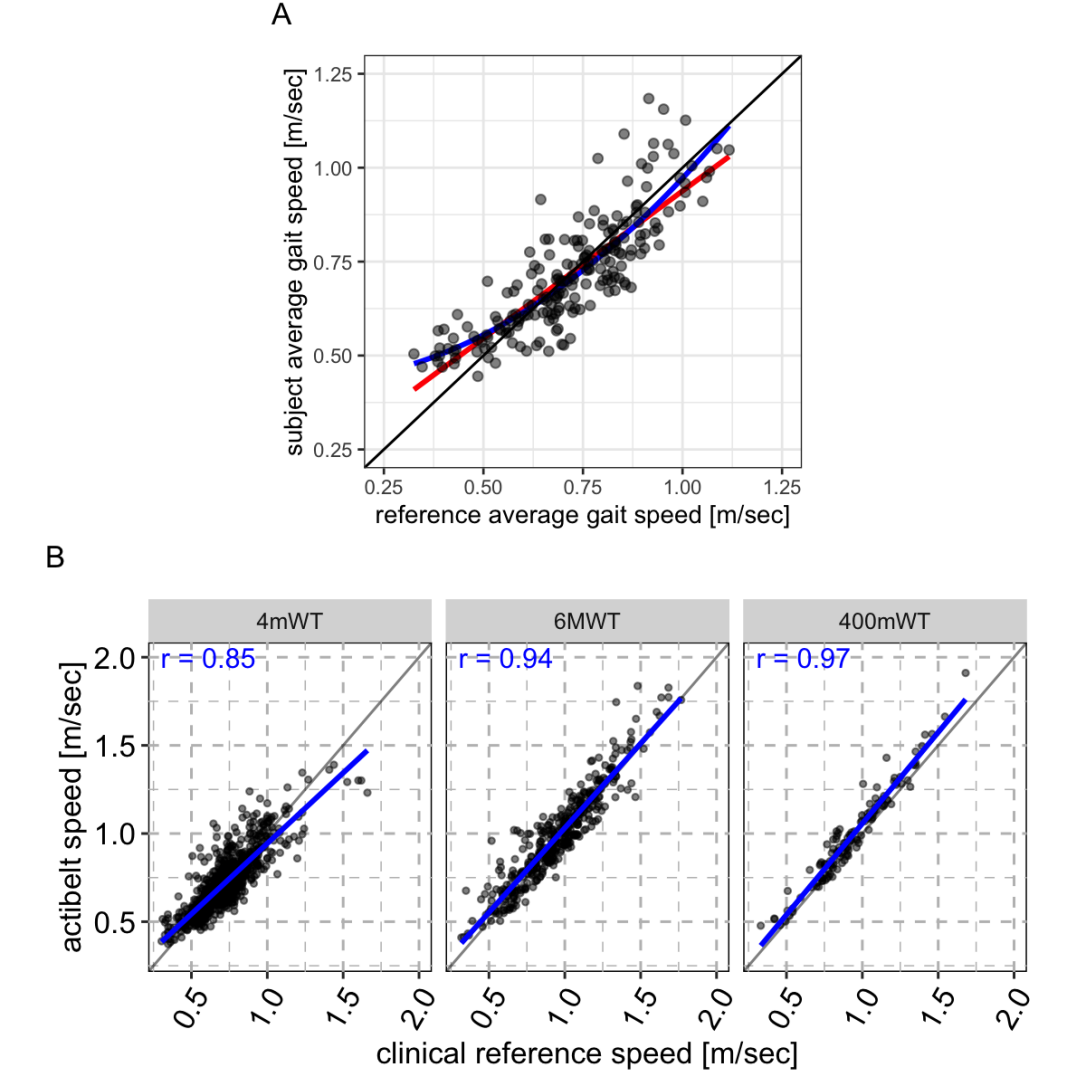
Accuracy of the algorithm in frail, slow walking adults. (A) Results from the independent validation study “parcours”. Reference gait speed continuously captured using an assistant-operated device is shown on the x axis, and accelerometer-derived patient gait speed is shown on the y-axis. Each datapoint represents the median speed for a given subject and parcours section. Derived gait speed is shown to strongly associate with reference gait speed in this parcours setting, the intercept for the linear fit (red line) is 0.15 and the slope is 0.78 the residual standard error is 0.08 m/sec. For comparison, a cubic fit is included (blue line). (B) Results from sarcopenic adults as captured during scheduled clinical walking test assessments in the interventional clinical trial. Reference gait speed (calculated as the distance traveled by the patient during the assessment divided by the time taken to complete the assessment) is shown on the x-axis, and accelerometer-derived gait speed is shown on the y-axis. Each datapoint represents the average speed for a given patient and assessment. The intercept for the linear fit is 0.15, 0.09, 0.09 from left to right and the slope is 0.79, 0.95 and 0.96, the residual standard error is 0.08, 0.09 and 0.07 m/sec for the 4mWT, 6MWT and 400mWT (panels, left to right), respectively. Note, the 400 meter walk test was collected for relatively few patients. A strong linear association is observed between derived and reference gait speed in all assessments indicating accuracy in our target population of frail, slow walkers.

As shown in Figure 1, we observed a strong association (r=0.84; residual standard error=0.08 m per second) between the reference and sensor-estimated gait speed across all subjects and walking environments (see also Supplementary Figure 1 in Multimedia Appendix 1). This demonstrates, for the first time, accuracy of gait speed capture by a single system across a wide range of nonsimulated gait speeds (<0.5 to >1 m per second) in a frail population.

The same system (hardware and algorithm) was deployed to monitor patients in a global, multisite phase 2b interventional clinical trial involving patients with age-related muscle loss and slow walking speed (see section Methods). To further evaluate the accuracy of the system, we conducted internal validation within the interventional clinical trial dataset by performing a head-to-head comparison of gait speed calculated from accelerometry data and gait speed captured using canonical, clinical standard assessments. The assessments included a 4mWT [13], a 6MWT [14], and a 400mWT [15] from which we derived walking speed. Using timestamps demarcating the specific periods (±2 seconds) in which the assessments were performed, we extracted those intervals from the aligned raw accelerometry data stream and calculated the average gait speed using the algorithm described in the Methods section. Comparing these values with the corresponding clinical reference values (Figure 1), we could confirm that the new algorithm performs with less than or equal to 0.1 m per second residual standard error in our target population, with a correlation of 0.85, 0.94, and 0.97 for 4mWT, 6MWT, and 400mWT, respectively. The relatively high error seen in the 4mWT is because of the very short nature of the assessment, typically only taking a few seconds to complete, which, in turn, gives a greater weight to any intrinsic human error in recording the start and end of the assessment. The accuracy of our approach can overcome these errors and enable clinicians, which previously relied on the 4mWT, to draw more accurate conclusions.

### Feasibility and Patient Compliance in Long-Term, Real-World Settings

The above results demonstrated the accuracy of both the hardware and algorithm for active, controlled-environment data collection. The monitoring of real-world behavior presents a more complex challenge because of dependence on patient compliance outside of the controlled clinic environment. We therefore set out to evaluate compliance and its effect on our ability to capture real-world walking behavior.

In the interventional clinical trial, patients were requested to wear the device at home for 5 days during the week before every scheduled visit to the clinical site. No feedback was directly given to patients, but if a patient reported that they had not worn the device during the week preceding a visit, they were requested to wear it for the following week. Compliance was monitored through regular data reviews, and feedback provided to clinical sites. We avoided providing any feedback on an individual patient basis so as not to influence their patterns of mobility and confound the overall trial. Ultimately, 9668 patient-days of accelerometry data were collected from 192 patients and 32 sites (see Supplementary Figure 2 in Multimedia Appendix 1 for patterns of observed compliance). A summary of enrolment and data collected is presented in Table 2. This demonstrates the feasibility of continuous monitoring in global clinical trial settings and elderly populations and enabled us to collect an unprecedented volume of data describing real-world mobility. We make the full raw and derived datasets for both the independent validation study and interventional clinical trial, publicly available as part of this publication (see section Methods for instructions on how to access).

We observed that patient compliance patterns were highly variable, with some patients greatly exceeding the requested wear time and others contributing far less. As previously reported [16], we sought to define a set of minimum thresholds for compliant wear time from which gait behaviors could be stably estimated. This 2-component threshold (hours per day and days around visits) is an attempt to maximize the number of patients included in the analysis and minimize variation because of the sampling of an unrepresentative, too short period of a patient’s daily life.

**Figure 2:**
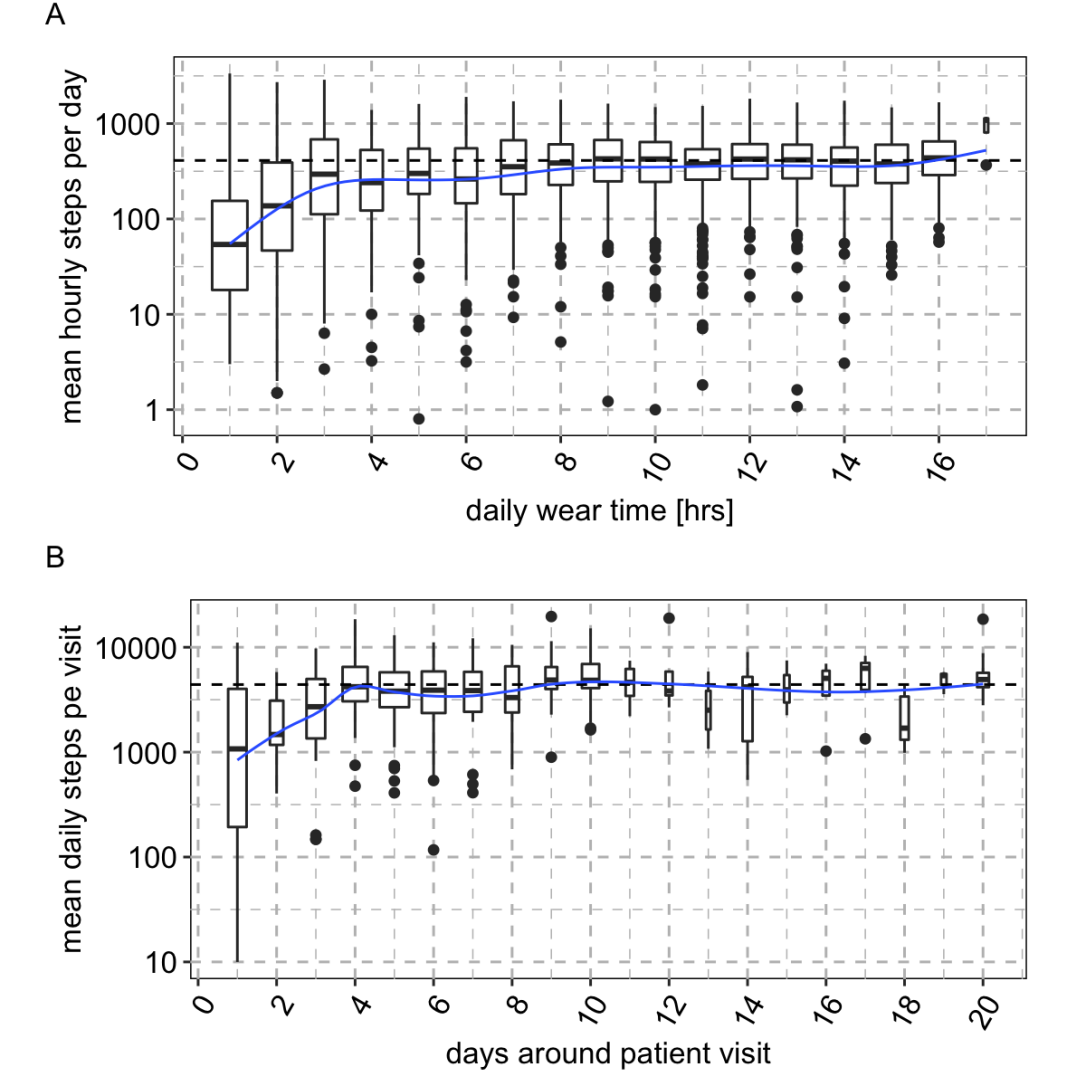
Effect of daily weartime and compliance around a visit on step count estimation. (A) The mean hourly steps per day is calculated for each day of patient observation as the total step count normalized to the detected weartime for that day. On the y-axis we show the distribution of mean hourly steps per day and patient, grouped by the total daily weartime in hours on the x-axis. The blue line is a smoothed Loess-fit. Mean daily steps are seen to drop sharply where less than 3 hours of weartime are detected. (B) After removing days of observation with less than 3 hours of weartime, we then calculated the distribution of average normalized daily step counts (“mean daily step count per visit”; total step count in a 20 day window straddling each planned visit divided by the number of compliant “days around patient visit” with a minimum 3 hours weartime). The “mean daily step count per visit” is plotted on the y-axis and the “days around patient visit” on the x-axis. The blue line is a smoothed Loess-fit. Mean daily steps per visit is observed to drop sharply where less than 3 compliant days are detected around a given visit. Combining the results of (A) and (B) we arrived at a two-component threshold of at least 3 days with at least 3 hours of compliance for robust capture of walking behavior in our population.

We extracted, for each patient, a 20-day period straddling each clinical site visit excluding the visit day itself. Figure 2 shows the total amount of daily wear time and daily total step count normalized to the total daily wear time. We expect the wear time–normalized daily step count to distribute around the grand mean (410 steps per hour, horizontal line); however, at less than 3 hours of wear time per day, the normalized step count decreases strongly, suggesting that, in our patient population, at least 3 hours of wear time on a given day is required for stable and representative sampling of walking behavior. Additional hours of wear time do not affect the normalized step count but reduce between-day step count variability as indicated by data comparing 3 versus 9 hours of daily wear time (Figure 2).

The patients in our study were asked to wear their belt with the accelerometer for at least five days around each scheduled clinical site visit, and this is reflected in the distribution of wear time in days around visits (Supplementary Figure 3 in Multimedia Appendix 1). However, some patients wore the belt for longer periods, and others for shorter periods. Figure 2 shows the mean daily step count when sampling for different number of days around visits. The normalized step count estimate starts to stabilize at 3 days in our population. Where a patient is only compliant for 1 or 2 days in the week of their visit, the variation is relatively high compared with longer sampling periods.

Combining these 2 observations, we chose a minimum criterion of at least three hours of wear time per day for at least three days per visit epoch for our real-world gait analysis. This enabled us to avoid biased estimates but also to minimize exclusion of patient visits and placed a very low burden on the patients. After applying these criteria, 398 visits from 160 patients remained from a complete dataset of 594 visits from 192 patients. This subset was used for all subsequent analyses and figures.

### Context Dependence of Real-World Gait Speed

Variability observed in real-world behaviors is heavily influenced by external factors [23]; for example, gait speeds observed in this study may be influenced by changing footwear or weather. Research is now beginning to show how overlaying external contextual information, for example, location [24], onto objectively captured sensor data and patient-reported outcome data can help explain some of the observed variations and enable even more meaningful comparisons. Although the wearable sensor that we deployed does not allow for the direct capture of contextual information, it has been shown that by grouping concerted periods of walking (*bouts*) by length, some context can be inferred and enable comparisons of distinct behaviors over time and between individuals [16,25,26].

We observed, on both the population and individual patient level, highly variable and skewed distributions in both bout length (Supplementary Figure 4 in Multimedia Appendix 1) and real-world gait speed (Supplementary Figure 5 in Multimedia Appendix 1).

**Figure 3:**
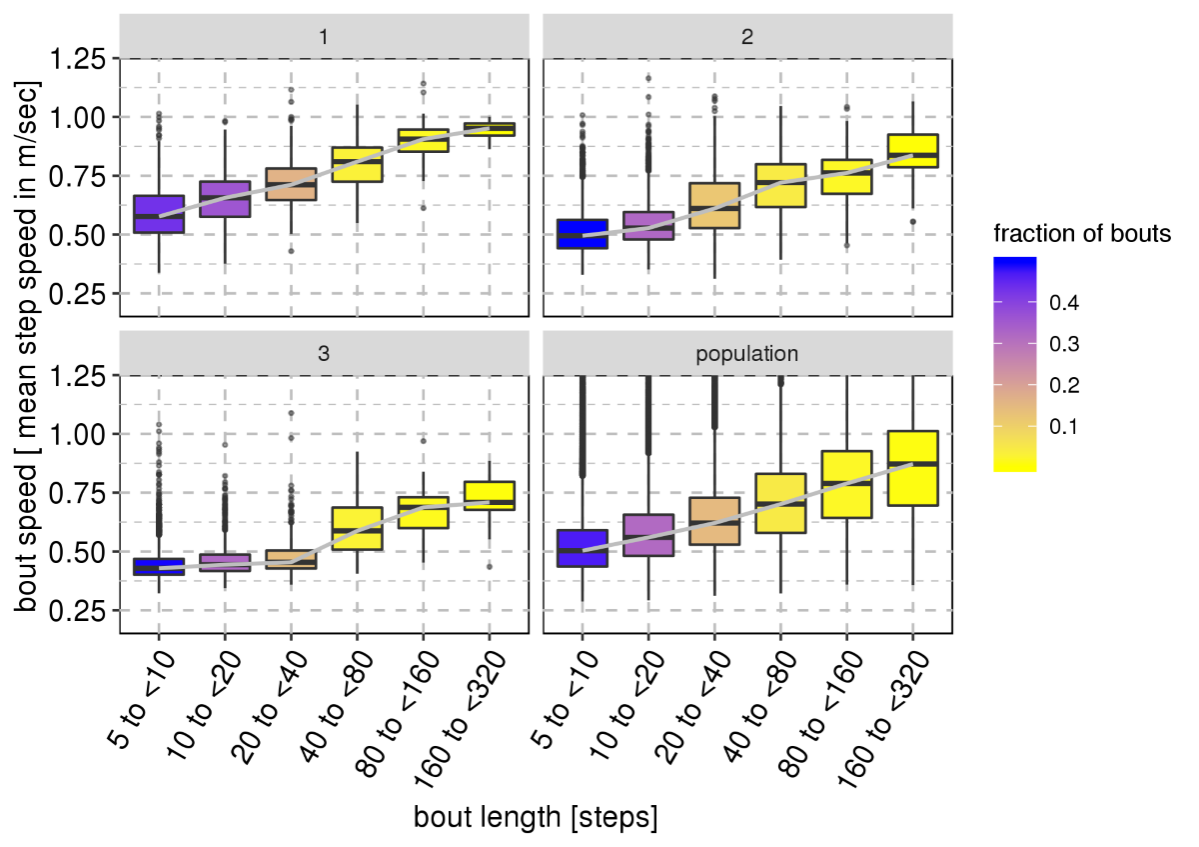
Comparison of gait speed with bout length on a population-level (bottom right panel) and for three representative individual patients (other panels). For all panels, the x-axis shows bout length, divided into groups of increasing numbers of steps, from very short bouts (fewer than 10 steps) to very long bouts (&lt;320 steps), and the y-axis shows the distribution of mean gait speed for each bout. Each boxplot is colored by the fraction of total bouts within that bout length range on a scale between dark blue (boxplots representing a large fraction of bouts) to light yellow (boxplots representing a small fraction of bouts). We observe that gait speed increases with bout length, and the majority of bouts are short in length (i.e. contain few steps).

The majority of observed real-world walking behavior comprised relatively short and slow bouts, although it is debatable whether some of these very short bouts of low acceleration intensity constitute true walking behavior. We examined the relationship between bout length and real-world gait speed and found that real-world gait speed strongly increases with bout length on both the population and individual patient level (Figure 3).

### Comparison of Gait Speed in Real-World Behavior and In-Clinic Performance

Understanding how real-world walking behavior is influenced by changing performance ability in clinical assessments is a key step in understanding how performance and subjective perceptions of independence are linked. Making comparisons between tests performed in the clinic and real-world behavior has proven more difficult, for example, in MS populations, and it was challenging to find sufficient *real-world* 6MWT events to make a meaningful comparison [4].

**Figure 4:**
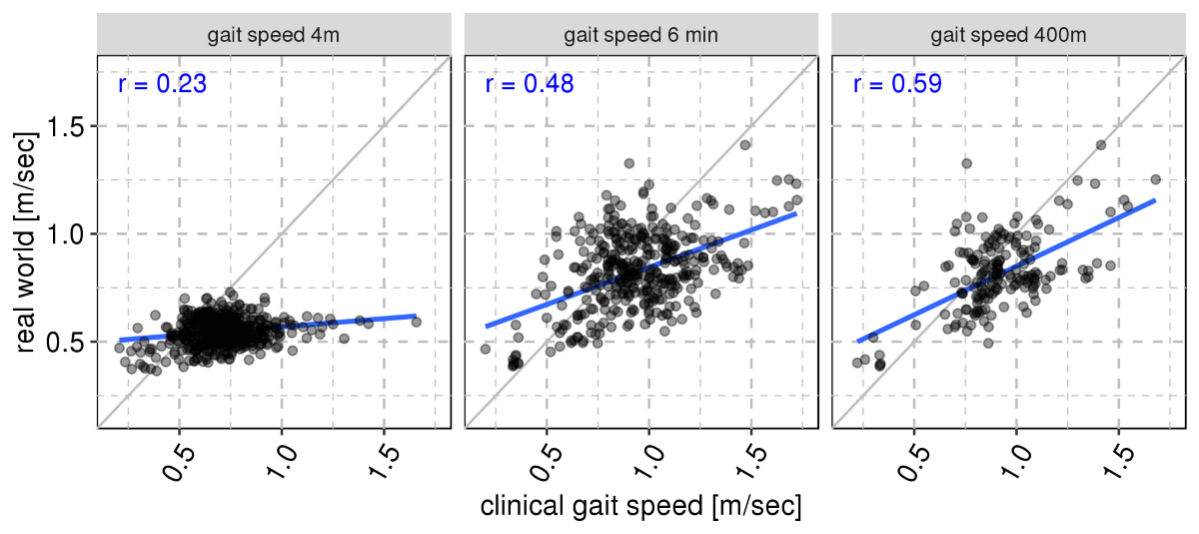
Comparison of in-clinic gait speed performance measures and adjacent real-world gait speed behavior. Only patient visits with at least 3 days and 3 hours per day of wearing in a 20 day window around the visit are included. Gait speed in the 4mWT is compared to real-world gait speed in bouts of length between 5 and &lt;20 steps, and 6MWT and 400mWT gait speeds are compared to real-world gait speed in bouts containing between 80 and &lt;640 steps. Gait speed in the clinical assessment is plotted on the x-axis and real-world gait speed is plotted on the y-axis.

We compared real-world gait speed from short (1-20 steps) and long (160-320 steps) bouts with the corresponding short (4mWT) and long (6MWT and 400mWT) clinical gait assessments. Applying our threshold for compliance, each comparison was made between a given clinical assessment and real-world gait from the 20 days surrounding that assessment, excluding the days of the assessment. We found strong linear relationships between real-world and in-clinic gait, in particular, for the longer gait assessments when comparing similar length bouts (Figure 4). Comparing the short 4mWT gait speed with gait speed in bouts between 5 and 20 steps, we saw a correlation of 0.23, and comparing the longer 6MWT and 400mWT results to bouts of 80 to 640 steps, we saw correlations of 0.48 and 0.59, respectively. Weaker associations were observed for comparison of nonsimilar bout lengths between the in-clinic assessments and real-world gait (Supplementary Figure 6 in Multimedia Appendix 1). Our results indicate that longer gait tests are the most reflective of real-world walking behavior.

We observed that real-world gait speed is consistently lower than what is observed in the corresponding clinic-based assessment. For example, a patient who records a 1 m per second 6MWT will, in their daily life, walk, on average, at 0.75 m per second for bouts of at least 80 steps. We found that this trend is reversed for the slowest walkers (below 0.5 m per second in clinical gait assessments), where the captured real-world gait speed is slightly higher. Overall, the positive associations between those measurements indicate that a higher mobility capacity is reflective of higher habitual gait speed in the real world.

## Discussion

### Principal Findings

We show, for the first time, accurate capture of real-world gait speed in slow-walking older adults with sarcopenia. In an independent validation study, recruiting 26 subjects with a mean gait speed of 0.62 m per second, we demonstrate a mean residual error of 0.08 m per second when estimating gait speed through a *parcours* course. Furthermore, using data captured from patients with sarcopenia with a mean gait speed of 0.648 m per second, as part of clinical gait assessments during an interventional clinical trial, we demonstrate residual standard errors of 0.08, 0.09, and 0.07 m per second for estimating gait speed during the 4mWT, 6MWT, and 400mWT clinical gait speed assessments, respectively.

We demonstrate the feasibility of long-term, real-world monitoring of gait and mobility in geriatric populations with slow-walking speed, capturing 9668 patient-days of accelerometry data from 192 patients and 32 sites. Our results establish that even in the absence of feedback or other incentives, sufficient data can be collected to allow robust monitoring of gait behaviors outside the clinic.

We indirectly infer context, in this case, bout length, to partly explain some of the large variation we see in real-world behaviors. Using this contextual information, we demonstrate the ecological validity of in-clinic gait assessments and explore the relationship between in-clinic performance and real-world behaviors relating to gait. We show that a linear relationship exists between real-world gait speed and clinical assessments, but only between comparable bout lengths.

### Strengths and Limitations

Improving our understanding of how a patient’s capacity, that is, what a patient *can* do, relates to their functional behavior, that is, what they *actually* do, is foundational to building a bridge between physiological changes induced by a given therapy and changes in a patient’s independence [27]. A more complete chain of evidence, incorporating physiological, functional, and subjective data, will enable development of interventions that genuinely improve patient HRQoL.

Our 2-component threshold for robust capture of real-world walking behavior, a minimum daily wear time of 3 hours and minimum period of 3 days, is well below our initial expectations and achievable in the vast majority of this population without any reminder system or other motivational tools. Moreover, this low threshold can still provide valuable insights into long-term mobility behaviors. Importantly, although relevant to this study population and protocol, this threshold may not be directly transferable to other settings without further evaluation. Other populations may differ in their wear time behavior and periodicity of activity; for example, in a working population, nonworking waking hours might be vastly different from working hours. We believe similar approaches could be modeled on what we present here to define these thresholds in the future populations and settings.

A recent study also compared distributions of real-world gait speed with 4mWT gait speed and found no association [28]. We find these results to be consistent with our own, as we see no relationship to overall distributions, but only find positive associations when comparing similar bouts between clinical assessments and real-world gait. In addition, we demonstrate that real-world gait was consistently slower than what was seen in the clinic, an observation also noted by Van Ancum et al [28], and reflecting other data showing that a patient’s gait speed can be increased simply by the knowledge that they are being observed [29].

### Outlook and Conclusions

Future work will focus on the clinical relevance and value of this novel continuous monitoring approach by examining changes over time and response to therapeutic intervention. We will also continue exploration of how context can enable interpretation of real-world behavioral data, for example, overlaying weather data from a patient’s locality to model seasonal changes in mobility [30]. These efforts may further explain variation in the real-world data and enable more sensitive longitudinal comparisons. Further stratification of bouts may be possible by combining information on location from anonymized global positioning system data [24] or gyroscope data to distinguish linear and nonlinear bouts.

Direct, accurate measurement of performance capacity and behavior specifically relating to physical activity is still an emerging field but holds the promise of a better understanding of how mobility, independence, and HRQoL are interrelated on an individual patient level. The work presented here is based on a relatively simple accelerometry-based system and still provides many advantages alongside traditional approaches for assessing mobility, yet capturing broader, multimodal data covering domains such as social interaction [31], stress [32], or vital signs could potentially increase our understanding of physical effort for a specific activity [33]. Directly capturing context, behavior, and activities of daily living using ambient [34] and smartphone technologies [24] will help us to better relate objective measurements to events and transitions in a patient’s life. Ultimately, we aim to build on the progress presented here in terms of establishing accuracy, feasibility, and leveraging context to make meaningful comparisons to further build the link between performance, behavior, subjective perceptions of health, and clinical outcomes [35] and to predict long-term changes in health.

## Appendix

Multimedia Appendix 1 Supplementary figures 1-6.

## Abbreviations

400mWT: 400 m walk test
4mWT: 4 m walk test
6MWT: 6-min walk test
HRQoL: health-related quality of life
MS: multiple sclerosis
