# Supplementary material for: Continuous Digital Monitoring of Walking Speed in Frail Elderly Patients: Noninterventional Validation Study and Longitudinal Clinical Trial
Source: JMIR Mhealth Uhealth. 2019 Nov 27;7(11):e15191. doi: 10.2196/15191 (PMC6906618; doi:10.2196/15191)
Supplement: Multimedia Appendix 1 [file mhealth_v7i11e15191_app1.docx]

Supplementary Materials


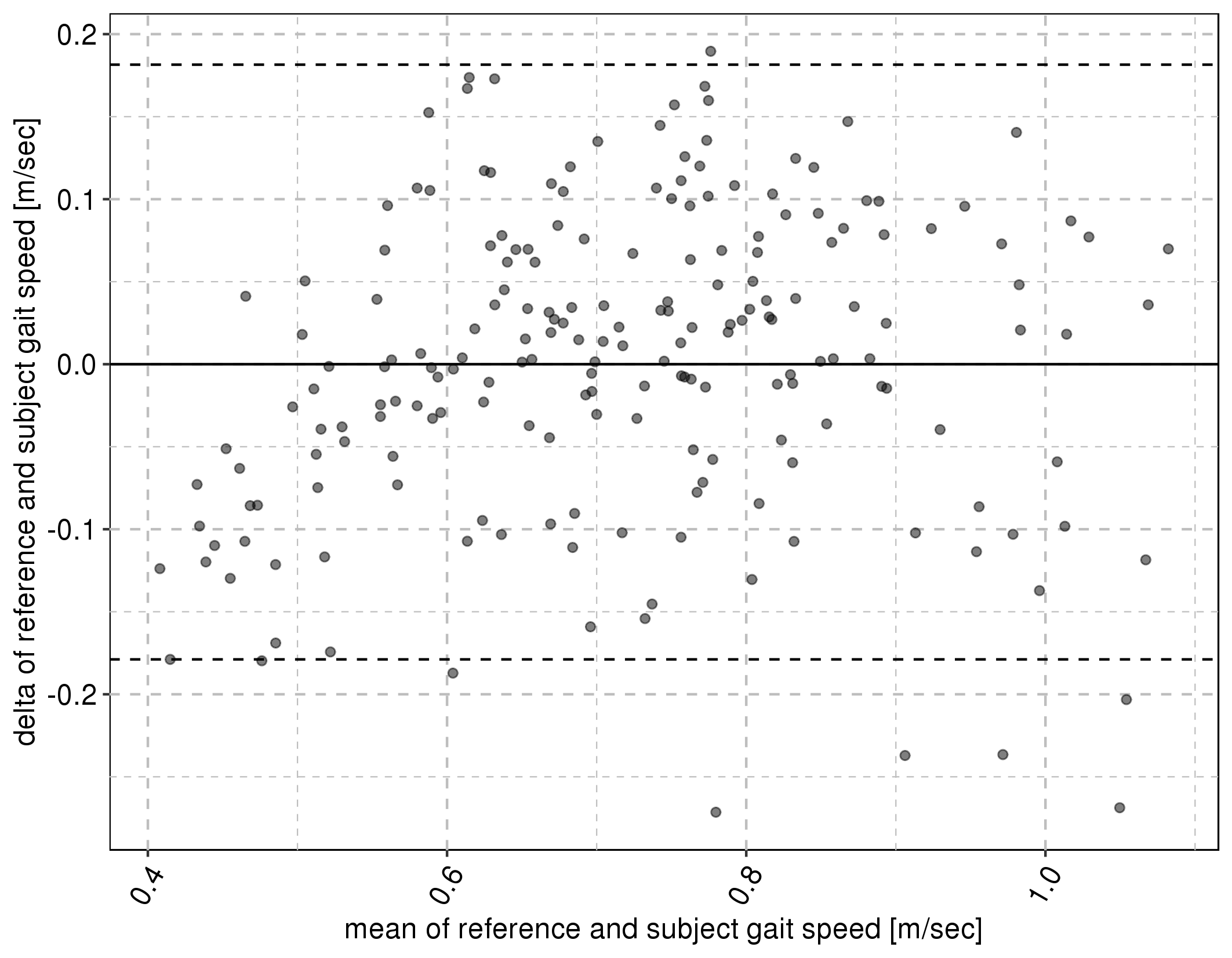


**Supplementary Figure 1.** Bland-Altman plot describing the mean of (x-axis), and difference between (y-axis), reference gait speed and accelerometry-derived gait speed captured during the independent validation study. Each point represents a given subject and parcours section. The solid horizontal line is the grand mean (0.001 m/sec) and the dashed lines indicate the 95% confidence interval.


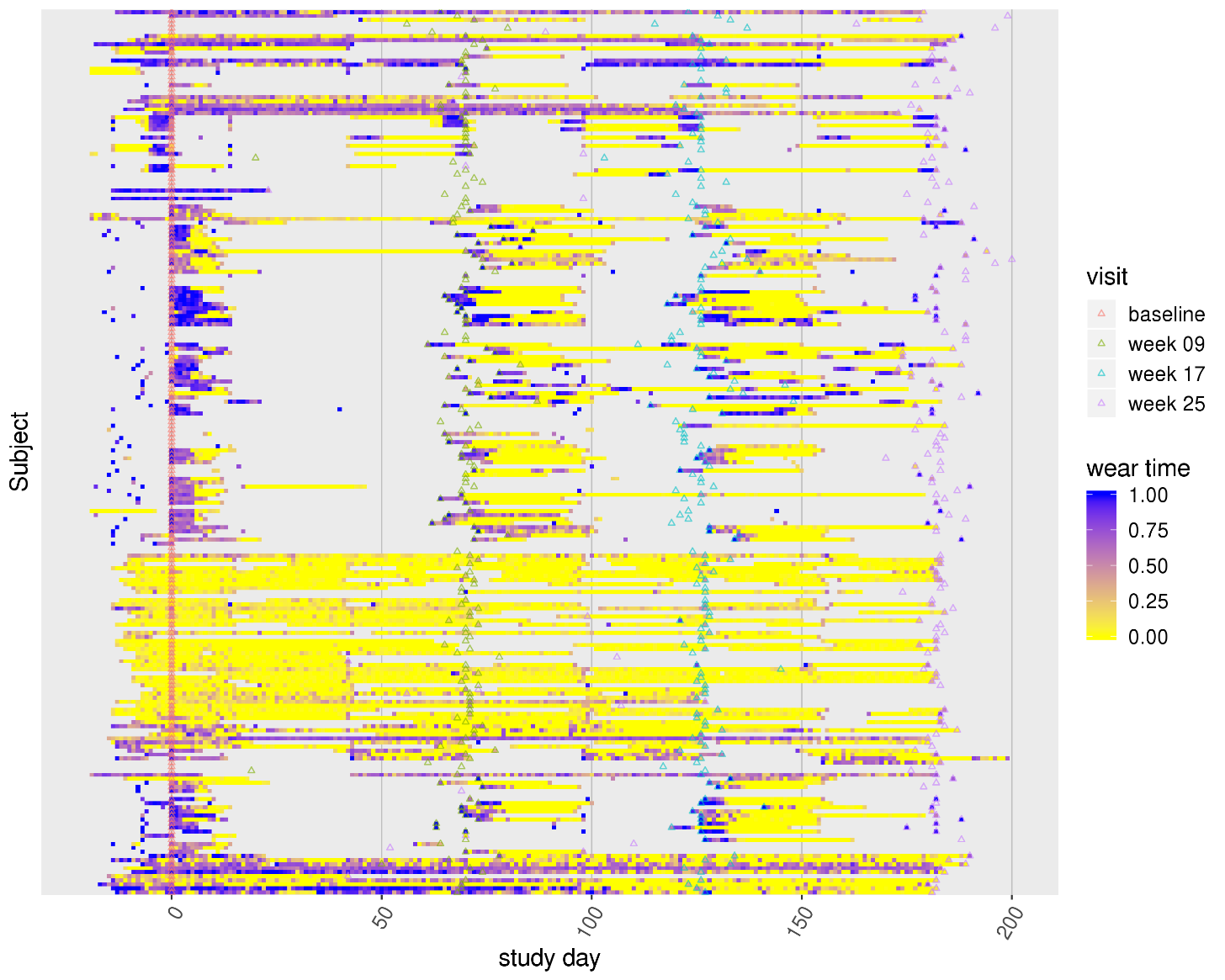


**Supplementary Figure 2**: Real-world compliance. The x-axis is ordered by study day relative to baseline (study day 0). The y-axis represents the datastream collected from a single patient as a single row, clustered by overall wear time. Compliance was measured as the fraction of daily weartime between 4 am to 8 pm, where 1 (represented as dark blue) equals complete compliance and 0 (represented as yellow) equals no weartime detected, with intermediate values represented on a continuous scale between these extremes, grey cells indicate periods when the patient did not have a belt at home. Visit days per patient are indicated as colored dots.


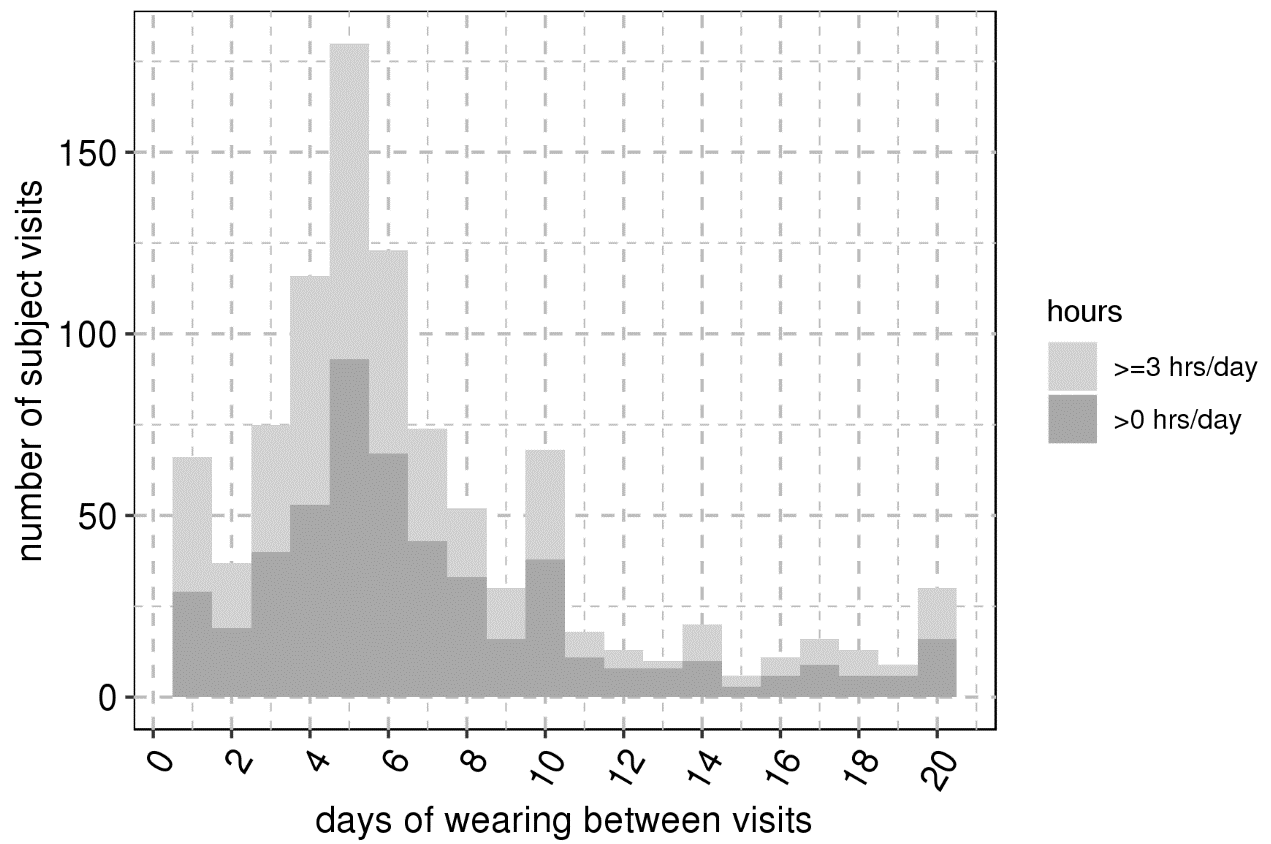


**Supplementary Figure 3**: Distribution of the number of weartime days per visit in the patient population. Dark grey is the subset of patients with at least 3 hrs or weartime per days and light grey with any wear time (> 0 hrs). Weartime per visit is the sum of days in a window of +/- 10 days around a visit. Note, that patients were asked to wear the device 5 days before each visit.


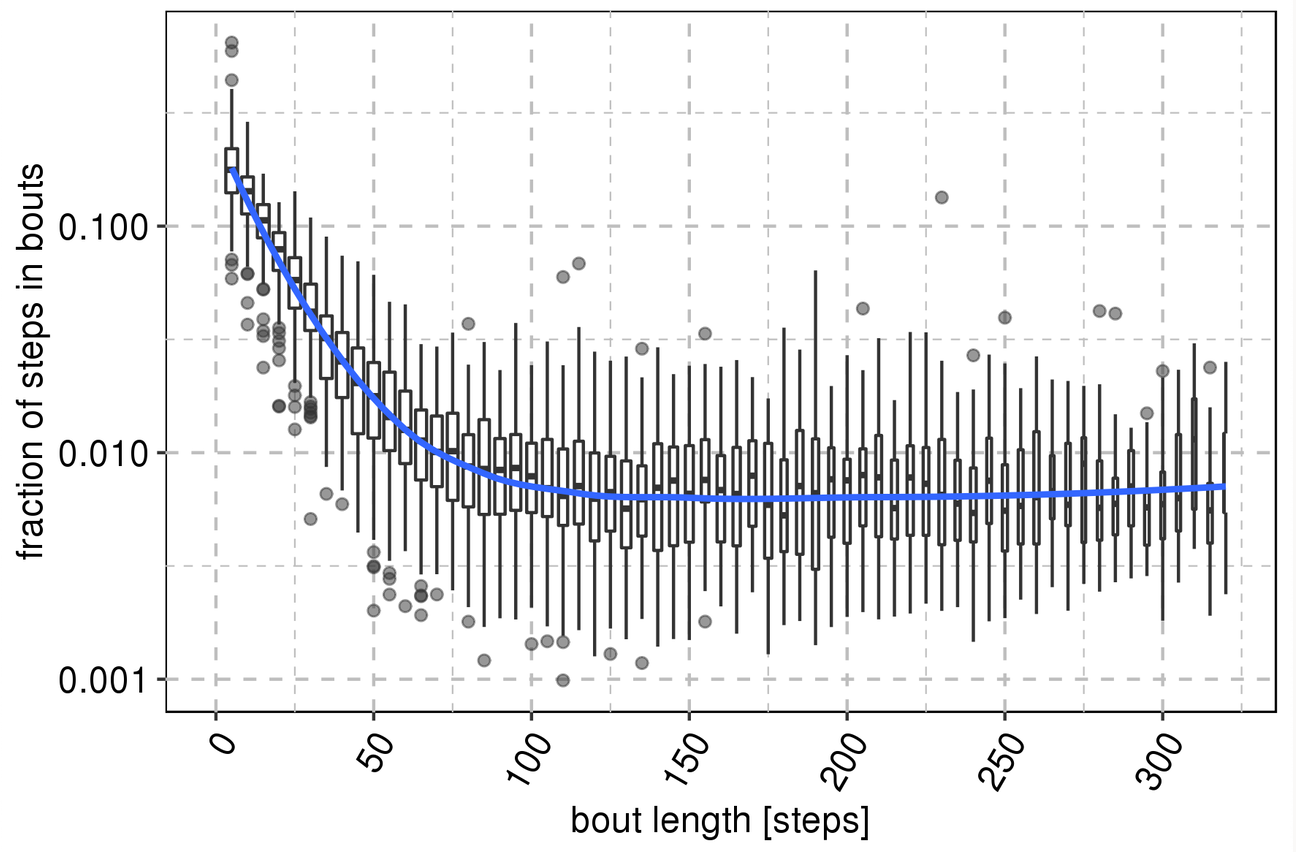


**Supplementary Figure 4**: Population-level distribution at baseline for observed real-world bout length. Each box is the frequency distribution (y-axis) for a bout length range with increments of 5 steps (x-axis, up to 320 steps in a bout) across subjects. The height of the boxes and whiskers give the variability between patients per bout length range, the width of the boxes is proportional to the number of observations (longer bouts are rare in our dataset). Note: we only include bouts of at least 5 steps to reduce the inclusion of shuffling non-walking behavior in our analysis.


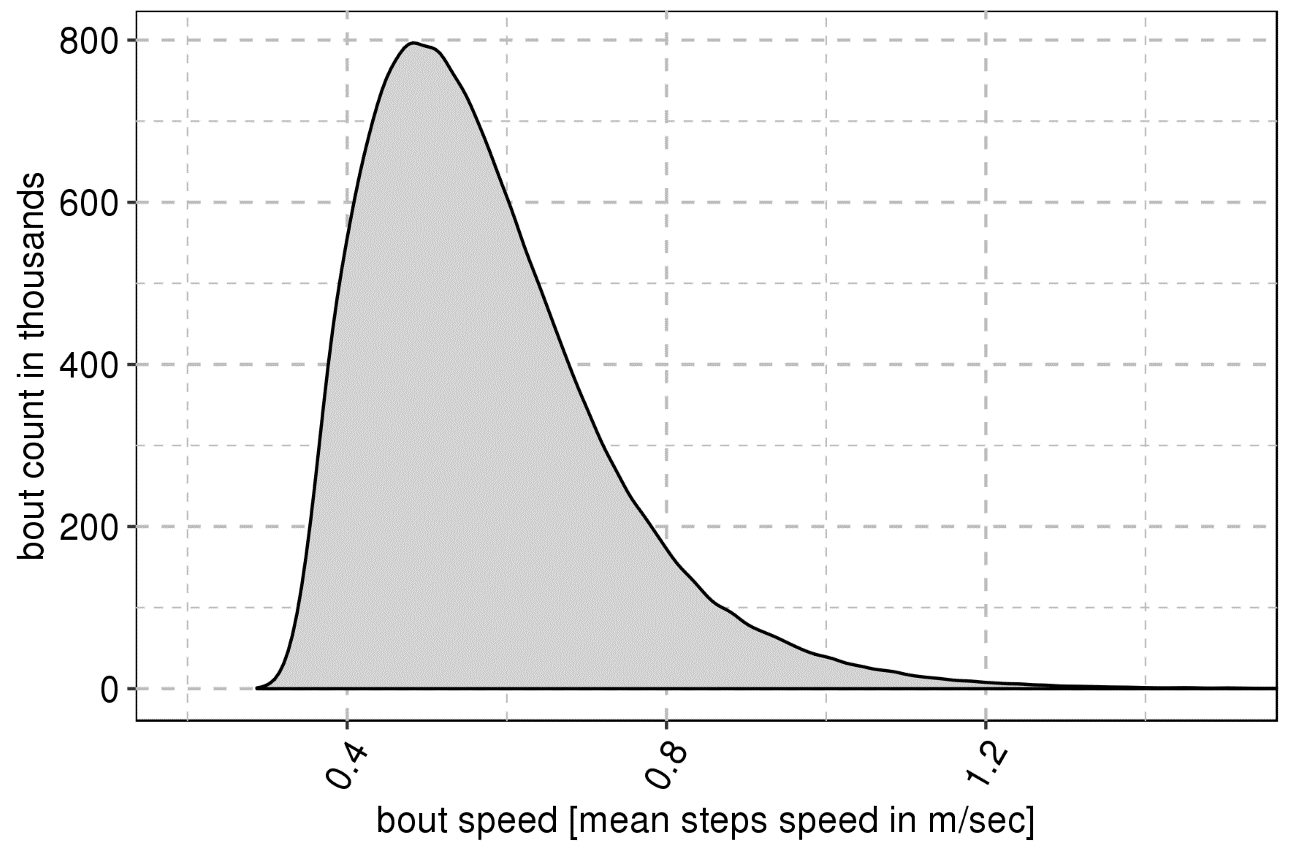


**Supplementary Figure 5**: Population-level, baseline distribution for observed real-world gait speed. Only bout lengths between 5 and 320 steps are included, as longer bouts are rare in our data set. The distribution is both highly variable and skewed, with relatively slow bouts making up the majority of observed real-world walking behavior.


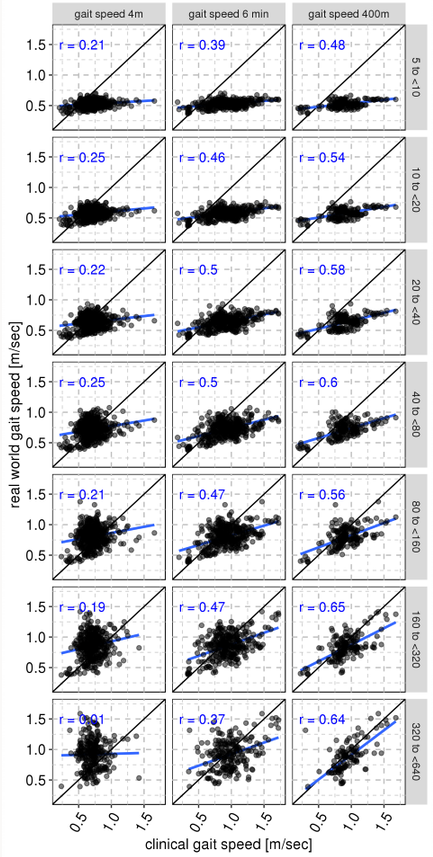


**Supplementary Figure 6**: Comparison of in-clinic gait speed performance measures and real-world gait speed behavior from the average of +/- 10 days around each visit (excluding the day of the visit). Comparisons are shown for short (4mWT) and long (6MWT and 400mWT) in-clinic tests (panel columns, left to right) and real-world gait speed observed in bouts of 5 to <10 steps, 10 to <20 steps, 20 to <80 steps, 80 to <160 steps, 160 to <320 steps and 320 to <640 steps (panel rows, top to bottom). In-clinic gait speed is shown on the x-axis and real-world gait speed on the y axis, Pearson correlation (r) is given in the top left corner of each panel.
